# PipY, a Member of the Conserved COG0325 Family of PLP-Binding Proteins, Expands the Cyanobacterial Nitrogen Regulatory Network

**DOI:** 10.3389/fmicb.2017.01244

**Published:** 2017-07-11

**Authors:** José I. Labella, Raquel Cantos, Javier Espinosa, Alicia Forcada-Nadal, Vicente Rubio, Asunción Contreras

**Affiliations:** ^1^Departamento de Fisiología, Genética y Microbiología, Universidad de Alicante Alicante, Spain; ^2^Instituto de Biomedicina de Valencia – Consejo Superior de Investigaciones Científicas Valencia, Spain; ^3^Group 739, Centro de Investigación Biomédica en Red de Enfermedades Raras – Instituto de Salud Carlos III Valencia, Spain

**Keywords:** cyanobacteria, PipX, COG0325, PipY, nitrogen regulation

## Abstract

*Synechococcus elongatus* PCC 7942 is a paradigmatic model organism for nitrogen regulation in cyanobacteria. Expression of genes involved in nitrogen assimilation is positively regulated by the 2-oxoglutarate receptor and global transcriptional regulator NtcA. Maximal activation requires the subsequent binding of the co-activator PipX. PII, a protein found in all three domains of life as an integrator of signals of the nitrogen and carbon balance, binds to PipX to counteract NtcA activity at low 2-oxoglutarate levels. PII-PipX complexes can also bind to the transcriptional regulator PlmA, whose regulon remains unknown. Here we expand the nitrogen regulatory network to PipY, encoded by the bicistronic operon *pipXY* in *S. elongatus*. Work with PipY, the cyanobacterial member of the widespread family of COG0325 proteins, confirms the conserved roles in vitamin B6 and amino/keto acid homeostasis and reveals new PLP-related phenotypes, including sensitivity to antibiotics targeting essential PLP-holoenzymes or synthetic lethality with *cysK*. In addition, the related phenotypes of *pipY* and *pipX* mutants are consistent with genetic interactions in the contexts of survival to PLP-targeting antibiotics and transcriptional regulation. We also showed that PipY overexpression increased the length of *S. elongatus* cells. Taken together, our results support a universal regulatory role for COG0325 proteins, paving the way to a better understanding of these proteins and of their connections with other biological processes.

## Introduction

Cyanobacteria are phototrophic organisms that perform oxygenic photosynthesis and require the assimilation of ammonia for autotrophic growth. This assimilation is carried out by the GS-GOGAT cycle, consuming 2-oxoglutarate (2-OG) (Muro-Pastor et al., 2001, 2005) which is a master regulator metabolite at the intersection between the carbon and nitrogen metabolic pathways, an thus an excellent indicator of the carbon to nitrogen balance (Muro-Pastor et al., 2001; Forchhammer, 2004; Laurent et al., 2005; Huergo and Dixon, 2015). 2-OG modulates the interactions of three key cyanobacterial proteins, two of which, the signal transducer PII and the global transcriptional regulator NtcA, bind 2-OG. The PipX protein can form alternative complexes with NtcA and PII and these interactions are, respectively, stimulated and inhibited by 2-OG, providing a mechanistic link between PII signaling and NtcA-regulated gene expression (Espinosa et al., 2006). PipX binds to either, 2-OG-bound NtcA to stimulate DNA binding and transcriptional activity, or to 2-OG-free PII to form PII-PipX complexes (Tanigawa et al., 2002; Vazquez-Bermudez et al., 2002; Llacer et al., 2010; Zhao et al., 2010; Forcada-Nadal et al., 2014), which in turn can bind to the transcriptional regulator PlmA (Labella et al., 2016). While PII is found in all three domains of life as integrator of signals of the nitrogen and carbon balance, PipX, NtcA, and PlmA are all restricted to cyanobacteria.

Knowledge of this nitrogen regulatory interaction network of cyanobacteria, has largely benefited from the “guilty by association” principle implicit in yeast two- and three-hybrid approaches (Burillo et al., 2004; Espinosa et al., 2006; Llacer et al., 2007, 2010; Laichoubi et al., 2011, 2012; Labella et al., 2016). The same principle can be applied to proteins encoded within the same operon, particularly in the cyanobacterium *Synechococcus elongatus* PCC7942 (hereafter *S. elongatus*), where most of the mRNA transcripts identified (approximately 62%) are monocistronic and thus the co-transcription of 2 given genes provides a very strong suggestion of functional association (Vijayan et al., 2011; Memon et al., 2013). This is the case of *pipX* and its downstream gene *Synpcc7942_2060*, called hereafter *pipY* in reference to its linkage to PipX in cyanobacteria.

**FIGURE 1:**
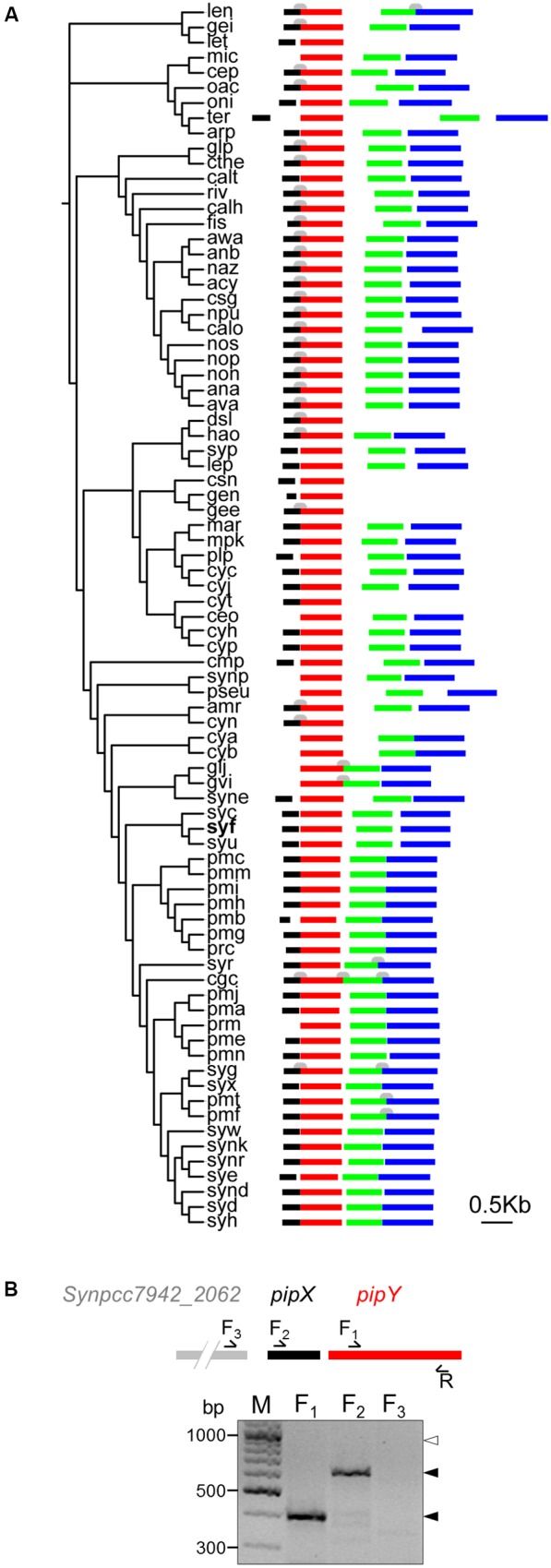
Genomic context of the *pipY* gene in cyanobacteria and co-expression of *pipX* and *pipY* in *S. elongatus*. **(A)** Left, phylogenetic tree of cyanobacteria genomes available in the KEGG database. Species names abbreviations are as in this database, with the one for *S. elongatus* in bold type. Right, schematic organization of the ORFs for *pipX*, *pipY*, *sepF*, and *proC* (black, red, green, and blue, respectively), with ORFs lengths and intergenic distances to scale. ORFs overlaps (1–56 bases) are indicated (gray shapes above ORFs; not to scale). In all cases genes are expressed from left to right. The thirteen species (can, cyu, mpro, scs, syj, syn, syq, sys, syt, syy, syz, tel, thn) having genomes in which *pipX* does not cluster with *pipY* are omitted. **(B)** Co-transcription of *S. elongatus pipX* and *pipY*. The scheme on the top shows the genomic region to scale, together with locations of the oligonucleotides used in RT-PCR assays (see Materials and Methods). The photograph at the bottom shows the PCR results for each of the indicated forward primers combined with the same reverse primer (R). Solid or open arrowheads indicate, respectively, the presence or absence of PCR products at the corresponding positions. M, DNA ladder, with band sizes (bp) given on the side.

Sequence comparisons indicate that PipY belongs to the COG0325 family, an intriguing group of highly conserved proteins that are widely distributed (Prunetti et al., 2016; and see *COG0325* in the EGGNOG database^1^). The reported structure of the COG0325 yeast protein (Eswaramoorthy et al., 2003) and the structures deposited in the Protein Data Bank (PDB), without associated publication, of the products of the *COG0325* genes of three additional microorganisms (*Escherichia coli*, *Agrobacterium tumefaciens*, and *Bifidobacterium adolescentis*; respective PDB files 1W8G, 3R79 and 3CPG) indicate that these proteins are single-domain pyridoxal phosphate (PLP) binding proteins that exhibit the fold type-III of PLP-holoenzymes (Percudani and Peracchi, 2009). Clustering of *COG0325* members with cell division genes in Gram-positive bacteria and mycobacteria suggested a connection with cell division in some bacterial groups (Gola et al., 2015; Prunetti et al., 2016). The genomic association of *pipY* with cyanobacterial-exclusive (*pipX*) and with more widely distributed bacterial genes (*sepF*) (Miyagishima et al., 2005), and the fact that in both cases synteny is observed in most cyanobacteria (illustrated in **Figure 1**), provide a strong motivation to investigate the role of COG0325 proteins in cyanobacteria in the context of paradigmatic bacterial processes such as nitrogen regulation or cell division.

Three studies addressing the characterization of *yggS* (encoding the *E. coli* COG0325 protein) null mutants revealed defects in amino/keto acid or vitamin B6 homeostasis (Ito et al., 2013; Prunetti et al., 2016). The pyridoxine sensitive phenotype of *yggS* null mutants could be complemented by heterologous expression of the plant and human COG0325 proteins, indicating that distantly related COG0325 proteins share common functions (Ito et al., 2013). Very recently, two different reports on human epilepsy (Darin et al., 2016; Plecko et al., 2017) confirmed the involvement of PROSC (the human COG0325 protein) in vitamin B6 homeostasis, showing that loss of function mutations at *PROSC* are a cause of vitamin B6-dependent epilepsy.

Although our initial interest in PipY stems from its predicted involvement in the cyanobacterial nitrogen regulatory network, in the course of this study the idea that COG0325 proteins may perform the same basic functions in all types of cells gained strength. Here we show that PipY is a *bona fide* member of the COG0325 family performing the same basic functions previously inferred for YggS and PROSC. Genetic analyses of *pipY* mutants (i) confirmed phenotypes observed in *E. coli yggS*, such sensitivity to pyridoxine and imbalance of the amino/keto acid pools (ii) revealed new PLP-related phenotypes, including sensitivity to antibiotics targeting essential PLP-holoenzymes or synthetic lethality with *cysK*, and (iii) uncovered gene interactions between *pipY* and *pipX*. We also showed that PipY overexpression increased the length of *S. elongatus* cells. Taken together, our results support a purely regulatory and universal role for COG0325 proteins, paving the way to a better understanding of these proteins and of their connections with other biological processes.

## Materials and Methods

### Construction of Plasmids, Cyanobacterial Strains, and Culture Conditions

Strains and plasmids used in this work are listed in **Table 1**. Oligonucleotides used to construct plasmids and for sequencing and strain verifications are listed in Supplementary Table S1. Cloning procedures were carried out with *E. coli* DH5α, using standard techniques (Sambrook et al., 1989). All the constructs were analyzed by automated dideoxy DNA sequencing. *S. elongatus* strains were routinely grown photoautotrophically at 30°C while shaking under constant illumination (40 μmol photons m^-2^s^-1^) provided by cool white fluorescent lights. Media used were blue–green algae media BG11 (BG11_0_ plus 17.5 mM NaNO_3_ and 10mM HEPES/NaOH pH 7.8) and BG11_A_ (BG11_0_ plus 5 mM NH_4_Cl and 5 mM HEPES/NaOH pH 7.8). For growth on plates the media was solidified by addition of 1.5% (w/v) agar. Plates were routinely incubated at 30°C under constant illumination. *S. elongatus* strains were transformed essentially as described by Golden and Sherman (1984).

**Table 1:** Strains and plasmids.

^∗^Strain or plasmid	Genotype, phenotype, relevant characteristics	Source
*Escherichia coli* DH5α	F^-^φ80 d*lacZ*ΔM15Δ(*lacZYA-argF*)U169 *endA1 recA1 hsdR17*(r_K_^-^ m_K_^+^) *deoR thi-1 supE44 gyrA96 relA1*λ^-^	Hanahan, 1985
*Saccharomyces cerevisiae* Y187	MAT*α ura3-52 his3-200 ade2-101 trp1-901 leu2-3, 112 gal4Δmet^-^ gal80Δ URA::GAL1_UAS_-GAL1_TATA_-lacZ*	Harper et al., 1993
*Saccharomyces cerevisiae* PJ696	MATa *ade*2Δ*trp*1-901 *leu*2-3,112 *ura*3-52 *his*3-200 *cyh*^R^ *can*^R^ *gal*4Δ*gal*80Δ *met*2^-^ *GAL*2::*ADE2 GAL1*::*HIS3* GAL7:*lacZ*	James et al., 1996
WT	*Synechococcus elongatus* WT	Pasteur Culture Collection
*pipX*	PipX^-^, Φ(P*_pipX_*::*cat*), Cm^R^	This work
*pipY*	PipY^-^, Φ(P*_pipX_*::*cat*), Cm^R^	This work
*pipXpipY*	PipX^-^ PipY^-^, Φ(P*_pipX_*::*cat*), Cm^R^	This work
*cysK*	CysK^-^, *cysK::Tn5*, Km^R^	This work
1^S^Ptrc	Φ(NSI-P*_trc_*), Sm^R^	Moronta-Barrios et al., 2013
1^A^Ptrc	Φ(NSI-P*_trc_*), Apr^R^	This work
1^S^Ptrc-PipY	PipY^C^, Φ(NSI-P*_trc_*::*pipY*), Sm^R^	This work
1^A^Ptrc-PipY	PipY^C^, Φ(NSI-P*_trc_*::*pipY*), Apr^R^	This work
1^S^Ptrc-PipX	PipX^C^, Φ(NSI-P*_trc_*::*pipX*), Sm^R^	This work
CK1X	PipX(Con) [Φ(C.K1–pipX)], Km^R^	Espinosa et al., 2006, 2010
*pipY*/1^S^Ptrc-PipX	PipY^-^ PipX^C^, Φ(P*_pipX_*::*cat*) Φ(NSI-P*_trc_*::*pipX*), Cm^R^ Sm^R^	This work
pBluescript Sk+	Cloning vector	Stratagene
pGAD424	*LEU2*, GAL4(768–881) AD (N-ter fusion), Ap^R^	Roder et al., 1996
pGBT9	*TRP1*, GAL4(1–147) BD (N-ter fusion), Ap^R^	Bartel et al., 1993
pADC	*LEU2*, GAL4(768–881) AD (C-ter fusion), Ap^R^	Millson et al., 2003
pBDC	*TRP1*, GAL4(1–147) BD (C-ter fusion), Ap^R^	Millson et al., 2003
pUAGC59	pBluescriptSK+ with *pipXY* genomic region, Ap^R^	Espinosa et al., 2006
pUAGC124	pGAD424 with *pipXY* genomic region, Ap^R^	This work
pUAGC129	pBluescript SK+ with *pipXY* genomic region, Ap^R^	This work
pUAGC125	pGAD424 with *pipXY* genomic region, Ap^R^	This work
pUAGC126	PipX^-^, Ap^R^, Cm^R^	This work
pUAGC127	PipY^-^, Ap^R^, Cm^R^	This work
pUAGC128	PipX^-^ PipY^-^, Ap^R^, Cm^R^	This work
pUAGC103	P*nblA::luxAB*, Cm^R^	Espinosa et al., 2007
pUAGC280	NSI, P*_trc_ lacI*, Ap^R^, Sm^R^	Moronta-Barrios et al., 2013
pUAGC873	NSI, P*_trc_*::*pipX lacI*, Ap^R^, Sm^R^	This work
pUAGC276	NSI, P*_trc_ lacI*, Apr^R^, Sm^R^	This work
pUAGC290	NSI, P*_trc_*::*pipY lacI*, Apr^R^, Sm^R^	This work
pUAGC294	NSI, P*_trc_*::*pipY lacI*, Sm^R^, Sm^R^	This work
pIJ786	Apramycin vector, Apr^R^, Ap^R^	http://streptomyces.org.uk/redirect/cassettes/pIJ786.embl
pUAGC878	pGBT9 with *alr* genomic region, Ap^R^	This work
pUAGC879	Alr^-^, Ap^R^, Nt^R^	This work
pUAGC11	GAL4AD:PII, Ap^R^	Burillo et al., 2004
pUAGC12	GAL4BD:PII, Ap^R^	Burillo et al., 2004
pUAGC471	GAL4AD:PipX, Ap^R^	Espinosa et al., 2010
pUAGC472	GAL4BD:PipX, Ap^R^	Espinosa et al., 2010
pUAGC739	GAL4AD:PipY, Ap^R^	This work
pUAGC740	GAL4BD:PipY, Ap^R^	This work
pUAGC745	PipX:GAL4AD, Ap^R^	This work
pUAGC746	PipX:GAL4BD, Ap^R^	This work
pUAGC747	PipY:GAL4AD, Ap^R^	This work
pUAGC748	PipY:GAL4BD, Ap^R^	This work
UGS-26-A-7	Tn5 transposon insertion in *cysK*, Cm^R^, Km^R^	Holtman et al., 2005

*^∗^Only fully segregated *S. elongatus* strains are listed*.

To construct the plasmids to inactivate *pipX* or *pipY* (Cyanobase gene ID *Synpcc7942_2061* and *Synpcc7942_2060*, respectively), the genomic region containing the *pipXY* cluster was excised from pUAGC59 with *Kpn*I/*BamH*I and cloned into pGAD424, yielding plasmid pUAGC124. In parallel, a 1.4 Kb genomic region, containing the operon sequences, was amplified using primers PipXXinact-R and PipXXinact-F and cloned into pBluescriptSK+, giving plasmid pUAGC129. A 1.3 Kb fragment was excised from pUAGC129 with *BamH*I and cloned into pGAD424, yielding plasmid pUAGC125. The coding sequence of the *cat* gene was PCR-amplified from pUAGC103 with primers pairs PipX-Cm-1F/1R and 2060-Cm-1F/1R containing homologous sequences to *pipX* and *pipY*, respectively. The *cat* PCR products bearing either *pipX* or *pipY* flanking sequences were used with linearized pUAGC124 (*Box*I) or pUAGC125 (*Sac*II), respectively, for recombination cloning in yeast, yielding plasmids pUAGC126 and pUAGC127.

Plasmids pUAGC126 and pUAGC127 were used, respectively, as templates for PCR amplifications with primer pairs ADCseq-F/pipX-Cm-1R and 2060-Cm-1F/GBT-2R. Both PCR products together with *EcoR*I/*Kpn*I restricted pGAD424 were used for recombination cloning in yeast, giving plasmid pUAGC128.

To overexpress *pipY* from the IPTG-inducible Ptrc promoter, two plasmids, pUAGC290 and pUAGC294, carrying P*trc::pipY* transcriptional fusion and *lacI* flanked by the Neutral Site I regions of *S. elongatus*, were engineered. The *pipY* sequences were PCR amplified from genomic DNA using primers ORF2060-4F and 2060-3R. The amplified product was restricted with *EcoR*I/*Sal*I and cloned into digested pUAGC280 and pUAGC276, yielding, respectively, plasmids pUAGC294 and pUAGC290. Plasmid pUAGC276 was obtained after amplification of DNA sequences conferring resistance to Apramycin with primers Apramicyn-1F/1R using pIJ786 as a template and the subsequent replacement of the Sm^R^/Sp^R^ cassette present in pUAGC280 by *Sph*I/*Hind*III digested Apramycin cassette.

To construct a *pipX* overexpression plasmid *pipX* coding sequence was PCR amplified from genomic DNA with primers pairs PipXOV2F/PipX-3R, restricted with *EcoR*I/*Bam*HI and cloned into digested pUAGC280 producing pUGAC873.

To engineer a plasmid to inactivate *alr* (*Synpcc7942_2201*), its genomic region was amplified with primers ALREcoRI-F/ALRBamHI-R and the product digested with *EcoR*I/*Bam*HI and cloned into restricted pGBT9 yielding plasmid pUAGC878. The coding sequence of *nat* gene was PCR-amplified with primers nat1ALR-F/nat1ALR-R containing homologous sequences to *alr*. The *nat* PCR product was used with restricted pUAGC878 (*Mls*I) for recombination cloning in yeast resulting in plasmid pUAGC879.

To inactivate *pipX*, *pipY*, or both simultaneously, WT *S. elongatus* was transformed with pUAGC126, pUGAC127 or pUAGC128, respectively. Transformants were selected with chloramphenicol (2.5 μg ml^-1^) and complete segregation was confirmed by PCR analysis with oligonucleotides pairs PipX126F/PipX5R, inter2060-1F/PipXXinact-R and PipX126F/2060Cm1F for *pipX, pipY* and *pipXY* inactivations, respectively. Digestion with *Tas*I was performed to differentiate WT (2 bands of ∼800 and 400 bp) and mutant (three bands of ∼500, 400, and 300 bp) alleles. For ectopic overexpression of *pipY* or *pipX* in *S. elongatus*, plasmids pUAGC290 and pUAGC294 for *pipY* and pUAGC873 for *pipX*, were transformed and clones were selected with either apramycin (10 μg ml^-1^) or streptomycin (5 μg ml^-1^). The correct insertion into the Neutral Site was confirmed by PCR with primer pairs PTRC99Aseq-F/NSI-1F and 7942NSIA-F/NSI-1R. For constitutive increased expression of *pipXY* operon in *S. elongatus*, pUAGC410 was transformed in WT and transformants selected with kanamycin (5 μg ml^-1^).

Inactivation of *cysK* (gene id *Synpcc7942_1466*) was carried out with plasmid UGS-26-A-7 from the unigene set, an arrayed mutant library for *S. elongatus* (Holtman et al., 2005). Transformants were selected on kanamycin plates (10 μg ml^-1^) and complete segregation confirmed by PCR analysis using primers cysK-1F/cysK-1R. To inactivate *alr* (gene id *Synpcc7942_2201*) pUAGC879 transformants were selected on plates with nourseothricin (5 μg ml^-1^) and segregation was analyzed with primers ALREcoRI-F/ALRBamHI-R.

To construct plasmids pUAGC739 and pUAGC740 for yeast two-hybrid interaction assays, the *pipY* coding sequence was amplified with primers PIXX-F and PIXX-R from genomic DNA, the product restricted with *EcoR*I/*Sal*I and cloned into digested pGAD424 and pBGT9. To obtain PipX and PipY derivatives carrying GAL4 (AD or BD) C-ter fusions, the *pipX* and *pipY* coding sequences were PCR amplified from genomic DNA with primers pairs pipX-ADC-F/pipX-ADC-R and pipX-BDC-F/pipX-BDC-R for *pipX* and pipY-ADC-F/pipY-ADC-R and pipY-BDC-F/pipY-BDC-R for *pipY*. PipX-ADC and PipY-ADC products were used as templates in a second round of PCR with primers ADC-F/ADC-R and similarly PipX-BDC and PipY-BDC products were amplified with BDC-R/BDC-R. ADC and BDC amplification products were cloned, respectively, into *Nru*I restricted pADC and pBDC yielding plasmids pUAGC745 and pUAGC746 for PipX, and pUAGC747 and pUAGC748 for PipY.

### PipX and PipY Immunodetection by Western Blotting in *S. elongatus*

For use as immunodetection standards, pure His_6_-tagged *S. elongatus* PipX (Llacer et al., 2010) and PipY (details to be published elsewhere) were used. This last protein was the antigen utilized for preparation of the primary antibody for PipY immunodetection (1.8 μg ml^-1^ IgG from anti-PipY rabbit antiserum, prepared by Genosphere Biotechnologies, Paris).

Luminescent immunodetection in western blots of homogenates of nitrate-containing liquid cultures grown to mid-exponential phase (OD_750_ ∼ 0.5) was carried out as previously reported for PipX (Labella et al., 2016). For PipX and PipY analysis a Precellys Evolution (Bertin Technologies) glass beads homogenizer was used for cell breakage (six 25-s 6,800 rev min^-1^ pulses separated by 1 min ice-cooling intervals).

### RT-PCR Analysis

RT-PCR assays were performed using 50 ng of DNase-treated total RNA, isolated as described (Lopez-Redondo et al., 2010) from ammonium growing cells. After retrotranscription of particular mRNAs, cDNA was used as template in PCR reactions. To retrotranscribe *pipX*, *pipY* and *sepF*, primers PipXQ82A-R, PipX-5R-X and 2059-R were used, respectively. The cDNAs were amplified with primer pairs PipXF38A-F/PipXQ82A-R (for *pipX*), PipX-4F/PipX-5R-X (for *pipY*) and 2059-F/2059-R (for *sepF*). 10 μL of the PCR reactions were loaded in a 1.5% agarose gel to visualize the amplified products. Housekeeping gene *rnpB* was used as a control to verify the same input of RNA using primers rnpB-R for retrotranscription and rnpB-F as forward primer. For co-transcription analysis of *pipXY* operon, total RNA was subjected to retrotranscription using the reverse primer PipX5R-X and the cDNA used as template in a PCR reactions using the same reverse and three forward primers PipXQ34A-F, qPCR2060 and Syn2060F annealing, respectively, within the coding region of *pipX*, *pipY* and *Synpcc7942_2062*.

### Transcriptomic Analysis

RNA was purified [phenol-chloroform extraction and TURBO DNase (Ambion) digestion] from two independent 120-ml ammonium-containing cultures (OD_750_ ∼0.7) of each *S. elongatus* WT and *pipX*, *pipY* or *pipXpipY* mutants. RNA was quantified spectrophotometrically. Its integrity (RIN > 8) was proven with the Agilent RNA 6000 Nano kit. mRNA enrichment from total RNA, cDNA library construction and sequencing were carried out with an Illumina’s TruSeq Stranded Total RNA with Ribo-Zero Kit and an Illumina Hiseq 2500 platform, using 100 bp paired-end sequencing reads.

Independent replicate datasets for the four *S. elongatus* strains, with entire genome coverage (all nucleotides covered) and 187–273 average deepness, were aligned with Bowtie2 (Langmead and Salzberg, 2012) with the sequence of the *S. elongatus* chromosome and endogenous plasmid (Genbank entries CP000100 and CP000101, respectively), retrieving expression values for each ORF with HTseq (Anders et al., 2015), followed by differential expression analysis with R package DESeq (Anders and Huber, 2010) and hierarchical clustering and dendrogram construction with R software (R Development Core Team, 2010) and the Ape package (Paradis et al., 2004).

### Pyridoxine (PN) and Antibiotic Susceptibility and Protection Assays in *S. elongatus*

The cells from 3-ml aliquots of the indicated *S. elongatus* strains grown to OD_750_ ∼0.6–0.7, suspended in 0.4 ml, were plated on solid medium supplemented when indicated with amino acids (6.75–225 μM for D-ala, 225 μM for L-amino acids) or pyridoxal (150 μM). A sterile disk filter was placed on the top of the plate, at the center, having been spotted with 7 μl of 21.5 mg ml^-1^ PN or with 4 μl of one of the following antibiotics (antibiotic/concentration in mg ml^-1^): apramycin/17.5, tetracycline/7, ampicillin/0.3, vancomycin/0.14, gentamycin/0.525, DCS/12.5, BCDA/12.5. After 3-days incubation at 30°C under constant light, the radii of the inhibition halos were measured. Plates were photographed over a white and red background, the last one to improve digital discrimination of biomass.

### Microscopy and Image Acquisition

Exponential growing cells (5 μL) were mounted on 1% low-melting point agarose pads for microscopy. The samples were observed and photographed with a Leica inverted confocal microscope (running under Leica Confocal Software version 2.61, Leica Microsystems) using the HCX PL APO 63X oil-immersion objective, numerical aperture 1.4. Filter specificities for cyanobacterial auto fluorescence analysis were as follow: ex633, TD 488/543/633, em665-700. Image capture conditions were 8 bits with a 1024 × 1024 resolution and 5× electronic zoom.

### Computational Methods

Intergenic distances between *pipX*, *pipY*, *sepF*, and *proC* were calculated using their positions in the cyanobacterial genomes available on the KEGG database. A phylogenetic tree was constructed using PipX, PipY, SepF and ProC concatenated protein sequences (in the given order) with the online tool Clustal omega^2^ with default parameters. When no orthologs were found in a particular genome for one or more of the four proteins, only those present were concatenated.

To measure cell length from confocal microscope images a homemade script was used. The cell measuring algorithm consisted on: (i) automatic image-blurring and thresholding parameters determination, where multiple cycles of blurring, thresholding and cell detection are computed to achieve the highest detected cells/total cells ratio; (ii) cell detection based on user input cell proportions (maximum and minimum area and length/width ratio) and shape complexity (hull area/contour area ratio) limits; (iii) determination of the rotated minimum area bounding rectangle, whose length and width are considered those of the cell.

ImageJ software was used to measure the radii of growth inhibition halos (ImageJ Macro is available on^3^). Color channels were separated and green and blue ones discarded. The image was thresholded with default parameters and subjected to a process of 3 cycles of dilatation, 73 cycles of erosion and 70 further cycles of dilatation to smooth the shape of the halo and to eliminate artifacts. The resultant image was adjusted to an ellipse and the diameter of the halo was calculated as the average of the major and minor axes of the ellipse minus the diameter of the disk filter.

### Yeast Two Hybrid Assays

Standard yeast culture and transformation procedures were used (Ausubel et al., 1999). To determine interaction patterns among selected proteins, expression from the reporter gene *lacZ* in PJ696/Y187 diploids was determined in X-Gal overlay high sensitivity assays as described (Burillo et al., 2004; Labella et al., 2016).

## Results

### Genomic Context of *pipY*, the Cyanobacterial Gene Encoding a Member of the COG0325 Family

To get insights into the genomic context of *pipY* in cyanobacteria, we retrieved homologous sequences from all cyanobacterial genomes available in the KEGG database^4^. In addition to *pipX*, two other genes, *sepF* and *proC*, were found clustering with *pipY* and were also included in the *in silico* analyses. The genomic context of *pipY* and the distances between contiguous ORFs are schematically illustrated within a phylogenetic tree constructed with the concatenated cyanobacterial sequences of these four genes (**Figure 1A**). Linkage between *pipX* and *pipY* is present in most cyanobacterial genomes, with very small or non-existent distances between the two ORFs: less than 167 nt in 71/94 cases, of which 29 show overlapping (1 or 4 nt). Thus, the genomic information indicates that co-expression and even translational coupling between *pipX* and *pipY* may be relatively frequent amongst cyanobacteria, suggesting a strong functional connection between PipX and PipY in this phylogenetic group. In the case of *S. elongatus*, our results from RT-PCR (**Figure 1B**) and those by others (Vijayan et al., 2011) indicate that *pipX* and *pipY* form a bicistronic operon.

In *S. elongatus* and in a majority of the available cyanobacterial genomes, the genes *sepF* [involved in cell division and restricted to gram positive bacteria and cyanobacteria (Hamoen et al., 2006)] and *proC* (Pyrroline-5-carboxylate reductase, EC 1.5.1.2) (De Wergifosse et al., 1994) were found at short distances downstream of *pipY*. These two genes have no paralogs in *S. elongatus*. Although *sepF* and *proC* are monocistronic in *S. elongatus* (Vijayan et al., 2011; Espinosa et al., 2014) the arrangements *pipY-sepF* and *sepF-proC* were also frequently found in the available cyanobacterial genomes, suggesting that co-transcription between *sepF-proC or pipY-sepF* may be frequent among cyanobacteria. The clustering of these 4 genes and the occasional overlap of *sepF* and *proC* ORFs (and, to a lesser extent between *pipY* and *sepF*) further suggest a functional linkage between the four genes. In this context, it is worth noting that synteny of *COG0325* members with both *sepF* and *proC* has been previously noted (Whitchurch et al., 1991; De Wergifosse et al., 1994). However, the precise arrangement *pipY-sepF-proC*, and the linkage to the nitrogen regulatory factor *pipX* are cyanobacterial hallmarks.

### Gene Inactivation and Polar Effects within the *S. elongatus pipXY* Operon

To study *in vivo* functions of PipY, constructs were engineered to inactivate the two genes of the *pipXY* operon, both individually and together. Gene inactivation was performed by allele replacement, precisely substituting the relevant coding region(s) by that of the *cat* gene (Supplementary Figures S1A,B). Subsequent detection by PCR and RFLP of fully segregated *pipY* null alleles indicated that *pipY* is not essential in *S. elongatus* (Supplementary Figure S1B). Growth curves of *S. elongatus* strains growing with either nitrate or ammonium as nitrogen source did not show significant differences between wild type (WT) and mutant strains (Supplementary Figure S1C), indicating that lack of PipY does not affect growth in either WT or *pipX* backgrounds.

**FIGURE 2:**
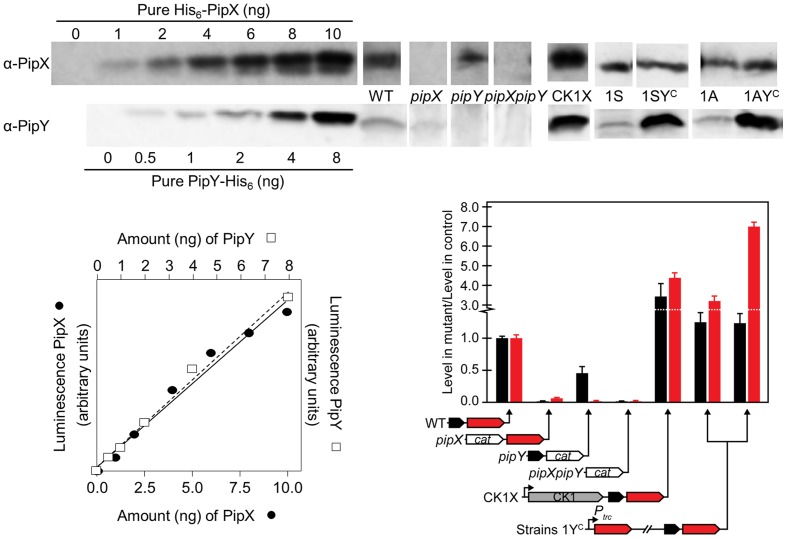
PipX and PipY protein levels in different *S. elongatus* genetic backgrounds. Illustrative examples of immunodetection signals for the indicated amounts of pure recombinant proteins (His_6_-PipX and PipY-His_6_) or for 25 μg (for PipX detection) or 50 μg (PipY detection) of total protein of the indicated extracts. Quantified luminescent signals with increasing amounts of the indicated recombinant proteins were plotted and the signal from extracts was interpolated in the calibration line (bottom left graph). Illustrative examples of PipX and PipY immunodetections in the different strains are shown. In each case, only the relevant region from the corresponding Western-blot were included. Specificity controls of the PipX and PipY immunoassays are provided by the corresponding null mutants. Histogram (bottom right) showing the levels of PipX (black) and PipY (red) proteins in the indicated strains relative to the corresponding levels in their control strain. The relevant chromosomal regions in the different strains are schematically illustrated below. The same representation (named 1Y^C^) applies to both 1^S^Ptrc-PipY (left) and 1^A^Ptrc-PipY (right) strains. CK1, kanamycin resistance cassette; *cat* chloramphenicol acetyltransferase gene. Strong promoters are indicated with black arrows.

We wondered whether inactivation of *pipX* or *pipY* affected transcript or protein levels from the reciprocal gene. RT-PCR showed decreased transcript levels of the non-inactivated gene of the *pipXY* operon, with *pipX* inactivation having a greater effect on *pipY* transcripts than the reverse (Supplementary Figure S1D). Since the transcript levels of *sepF*, used as an internal control, were not altered, the polar effects appear restricted to the *pipXY* operon. Importantly, Western blots showed that individual inactivation of *pipX* or *pipY* decreased the levels of PipY or PipX by 15-fold or 2-fold, respectively (**Figure 2** and **Table 2**), suggesting that the protein levels of PipY are highly dependent on *pipX* gene or gene product(s). In line with this, expression from the strong CK1 promoter (strain CK1X), resulting in a significant increase in the levels of PipX (Espinosa et al., 2010), produced a 3.4-fold and 4.3-fold increase in PipX and PipY, respectively (**Figure 2**).

**Table 2:** Protein levels and vitamin B6 related phenotypes of *S. elongatus pipX*, *pipY*, and *pipXpipY* strain.

Strain	^1^Protein levels		PN sensitivity		DCS sensitivity		BCDA sensitivity	
	PipX	PipY		+ PL		+ D or L-Ala		+ D or L-Ala
WT	126 ± 4	70 ± 4	+	+	+	-	-	-
*pipX*	0 ± 1	4 ± 1	ND	ND	++	-	++	-
*pipY*	57 ± 13	0 ± 1	++	+	+++	+	+++	-
*pipXpipY*	0 ± 1	0 ± 1	ND	ND	++++	+	++++	-

^1^*As mass (ng mg^-1^). Sensitivity to PN, DCS or BCDA is indicated in a qualitative scale from no (-) to high (++) sensitivity*.

### Phenotypic Analysis of *S. elongatus pipY* Mutants Reveals Universal Functions of the COG0325 Family

Given the singular genomic context of the *pipY* gene, it was important to show that *S. elongatus* PipY is a *bona fide* member of the COG0325 family performing functions previously proposed or shown for other members of the family. In this context, we tried to confirm in *S. elongatus pipY* the relatively simple phenotypes reported for *E. coli*, as well as to explore additional phenotypic differences between mutant and WT strains of *S. elongatus* based in the already known structural and/or functional features of COG0325 members.

**FIGURE 3:**
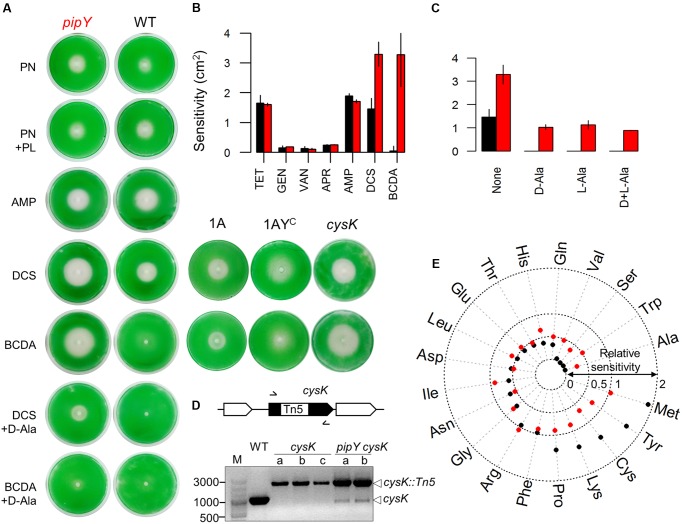
Phenotypic features of the *S. elongatus pipY* mutant. **(A)** Representative images illustrating the sensitivity of WT (black color throughout the Figure) and *pipY* (red) strains to the following compounds: PN (in the absence or presence of PL), DCS or BCDA (in the absence or presence of D-Ala), and AMP. **(B)** Sensitivity levels for the individual antibiotics shown as bar histogram of means and standard deviations (SD) for at least two independent experiments. **(C)** Effect of D-Ala (0.1125 mM), L-Ala (0.1125 mM) or both (0.1125 mM each) on the sensitivity of WT and *pipY* to DCS (Mean ± SD, at least 2 replicates). **(D)** Complete and incomplete inactivation *cysK* in WT and *pipY* backgrounds, respectively. Schematic representation of the relevant *S*. *elongatus* region, with indication of the positions of primer pairs (arrows), and PCR analyses of up to three independently transformed clones are show. Reference size bands and amplified alleles are shown to the left and right, respectively. **(E)** Radial plot illustrating the influence of adding individual proteogenic L-amino acids to the culture medium on the sensitivity to DCS, expressed as the sensitivity to DCS with/without the indicated amino acid. Dotted rings indicate total protection (0), half protection (0.5), no protection (1) and twofold increase of sensitivity (2) by the corresponding amino acid. Compounds concentrations are indicated in Section Materials and Methods.

We first tested whether the pyridoxine (PN) sensitivity phenotype of the *E. coli yggS* null mutant and its rescue by pyridoxal (PL) or by certain L-amino acids (Prunetti et al., 2016), could also be shown in the *S. elongatus pipY* mutant by disk diffusion assays on plates. As shown in **Figure 3A** and **Table 2**, the *pipY* mutant was significantly more susceptible to PN toxicity than the WT strain while addition of PL decreased PN toxicity in *pipY* mutants and not in WT.

Next, we tried to obtain evidence of a connection between PipY and the activity of PLP-holoenzymes, since a main consequence of COG0325 deficiency appears to be the low activity of PLP-holoenzymes (Darin et al., 2016; Prunetti et al., 2016; Plecko et al., 2017). To this end, we determined whether *pipY* inactivation affects the sensitivity to antibiotics targeting key PLP-holoenzymes such D-cycloserine (DCS) and β-chloro-D-alanine (BCDA), whose main target in bacteria is alanine racemase, an essential activity required for the synthesis of the cell wall (Feng and Barletta, 2003). To quantify the sensitivity of *S. elongatus* to antibiotics, we first confirmed the validity of using the square of the radius of the inhibition halo produced by different additions of antibiotic (Koch, 1999) using DCS in both WT and *pipY* strains. A good linear correlation between sensitivity and the amount of antibiotic added (Supplementary Figure S2) was found, thus validating the assays carried out here.

As shown in **Figures 3A,B**, and **Table 2**, the *pipY* mutant was more sensitive than WT to DCS and BCDA but not to control antibiotics that do not target PLP-containing proteins, including the protein synthesis inhibitors apramycin, tetracycline and gentamycin and the cell wall synthesis inhibitors ampicillin and vancomycin. Thus, the antibiotic sensitivity observed in the absence of PipY was very specific. To test if overexpression of PipY protects against DCS and/or BCDA we constructed plasmids and strains to increase PipY protein levels either constitutively or after IPTG induction (Supplementary Figure S3A). *S. elongatus* strain 1^A^Ptrc-PipY carrying P*trc*::*pipY* transcriptional fusion (**Figure 3A**, strain abbreviated as 1AY^C^) into the Neutral Site I (NSI) was tested alongside control strain 1^A^Ptrc (**Figure 3A**, strain 1A). The 7-fold increase in PipY levels found in strain 1AY^C^ (see **Figure 2** for details) impaired growth slightly (data not shown) and resulted in rather diffuse halos, but did not increase resistance to DCS or BCDA (**Figure 3A**). Addition of D-Ala rescued the sensitivity of *pipY* to BCDA and to a lesser extent to DCS (**Figure 3C** and **Table 2**), a result in line with the idea that BCDA is preferentially targeting alanine uptake and metabolism. Although D-Ala is known to be more effective against DCS than L-Ala in different bacteria (Wargel et al., 1970; Prosser and de Carvalho, 2013), we found that both amino acids added individually were equally effective protecting *S. elongatus* and do not show additive effects when added together (**Figure 3C**).

*Synechococcus elongatus* encodes 41 PLP-binding proteins (Percudani and Peracchi, 2009), including a putative alanine racemase, encoded by the *alr* gene (*Synpcc7942_2201).* To exclude the possibility that PipY could provide some alanine racemase activity in *S. elongatus*, we next tried to inactivate the *alr* gene in WT, *pipY* and 1^A^Ptrc-PipY strains. As expected, null *alr* alleles could not be completely segregated in either the WT strain or when PipY was expressed at higher levels, as in the 1^A^Ptrc-PipY strain (Supplementary Figure S3B), indicating the inability of PipY to complement Alr deficiency.

The most abundant PLP-binding proteins under standard culture conditions (Guerreiro et al., 2014) are PipY and a putative cysteine synthase (EC 2.5.1.47) encoded by *cysK* (*Synpcc7942_1466*), a non-essential gene (Rubin et al., 2015) with 3 paralogs in *S. elongatus*. Our attempts to inactivate *cysK* were successful in WT but not in *pipY* backgrounds (**Figure 3D**), indicating synthetic lethality between *pipY* and *cysK*, that is, both proteins contribute to essential cell functions, most likely related to the presence of the PLP cofactor. The *cysK* mutant was more sensitive than WT but less than the *pipY* mutant to both DCS and BCDA (**Figure 3A**), indicating that PipY and to a lesser extent CysK proteins behave as non-essential targets for antibiotics DCS and BCDA in *S. elongatus*.

To get further insights into PipY functions in the context of amino acid-related homeostasis, the effect of addition of each of the 20 L-amino acids on the susceptibility to DCS was determined for WT and *pipY* strains. The results are summarized as a radial plot in **Figure 3E**. Most amino acids altered the sensitivity to DCS, indicating that they entered the cell and/or transmitted signals with metabolic consequences. The exceptions were Arg and Phe, that did not alter DCS sensitivity in any of the two strains (red and black dots coinciding with a sensitivity ratio of 1 in **Figure 3E**), and here we cannot exclude failure of these amino acids to enter the cells in our experimental conditions. Remarkably, a previous study of amino acid uptake suggested very different permeabilities for these two amino acids in *S. elongatus*: while Arg uptake was negligible, Phe uptake was the highest amongst the 13 amino acids tested (Montesinos et al., 1997).

Four amino acids (Ala, Trp, Ser, and Val) exerted very large or complete protection against DCS in WT (black dots in **Figure 3E**) and incomplete protection in the *pipY* mutant (red dots in **Figure 3E**), indicating that a significant part of their protective effect is dependent on PipY. Nine amino acids (Gln, His, Thr, Glu, Leu, Asp, Ile, Asn, Gly) reduced but did not abolish growth inhibition by DCS to similar degrees in both strains (black and red dots near or outside the 0.5 ring but within the 1 ring). Another five amino acids (Pro, Lys, Cys, Tyr and Met) increased to different extents the sensitivity to DCS of the WT strain, while, with the exception of Met, they reduced somewhat the sensitivity of the *pipY* mutant, indicating that part of their toxic effect in the presence of DCS is dependent on PipY. The finding that the addition of certain amino acids produces opposite effects in WT and *pipY* strains of *S. elongatus* supports a complex role of PipY in amino acid homeostasis and calls attention to the diverse effects that different amino acids and metabolites can have on bacterial survival to DCS, a question of clinical relevance.

### Genetic, Not Necessarily Physical, Interactions between PipY, and PipX Revealed by Analysis of the Susceptibility of Mutants to DCS and BCDA

**FIGURE 4:**
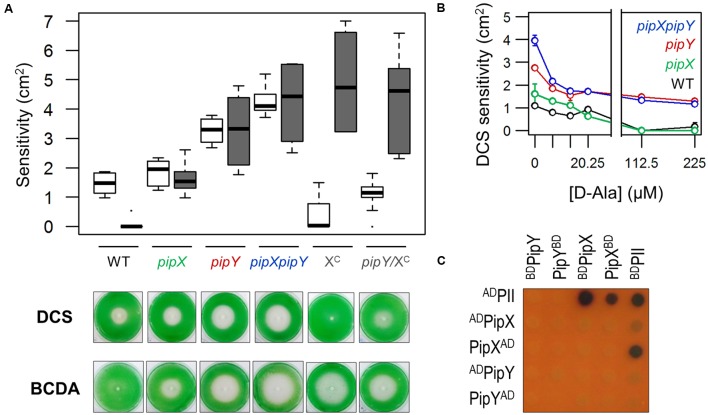
Interactions between PipY and PipX **(A)** *Top*, boxplot (median, box from first to third quartile, Tukey whiskers and outliers represented by dots) of DCS (white) and BCDA (gray) sensitivities for the indicated strains. In each case, a minimum of 3 independent experiments with 2 or more replicates were performed. *Bottom*, representative photographs illustrating the sensitivity of the indicated strains to DCS and BCDA. **(B)** Effects of different concentrations of D-Ala on the sensitivity to DCS (Mean ± SD, 2 replicates). **(C)** Representative example of yeast 2-hybrid interaction assays between the indicated proteins (see text for details). Yeast diploids were supplemented with X-gal to inform on *lacZ* reporter after 4 days of incubation on replete medium.

Once we obtained *in vivo* evidence indicating that PipY is a typical COG0325 protein, functioning in amino/keto acid and vitamin B6 homeostasis, we explored the PipY connection with the nitrogen regulator PipX in the context of the susceptibility to PLP-targeting antibiotics. To this end, we determined the susceptibility of *pipX* and *pipXpipY* mutants to DCS, BCDA and other antibiotics used as controls. As shown in **Figure 4A**, inactivation of *pipX* increased sensitivity to at least BCDA in WT (compare WT and *pipX*) and to at least DCS in *pipY* backgrounds (compare *pipY* and *pipXpipY*) without affecting sensitivity to the control antibiotics (Supplementary Figure S4), thus revealing gene interactions between *pipX* and *pipY*.

The greater DCS sensitivity of *pipXpipY* versus *pipY* suggested that PipX can protect against DCS in a PipY independent manner. To obtain additional evidence for this, we determined the effect of increasing PipX levels in the absence of PipY. To this end, we previously constructed 1^S^Ptrc-PipX (abbreviated as X^C^) and *pipY*/1^S^Ptrc-PipX (abbreviated as *pipY*/X^C^) strains (Supplementary Figure S3C). Suppression of the DCS hypersensitivity by overexpressing PipX and no significant effect on the BCDA phenotype (**Figure 4A**) indicated that an excess of PipX compensates for the lack of PipY in the presence of DCS, but not in the presence of BCDA. Moreover, addition of D-Ala allowed complete protection against DCS in WT and *pipX* strains and partial protection in *pipY* or *pipXpipY* strains (**Figure 4B**). Where complete or partial protection by D-Ala was obtained, protection was already significant at concentrations of D-Ala as low as 6.75 μM, and maximal at concentrations of 112–225 μM. Therefore, some PipY protein appears to be required for the protective effect of D-Ala (**Figure 4**) or L-Ala (**Figure 3**) in the presence of DCS, a result emphasizing the metabolic imbalance of the *pipY* mutant.

To investigate whether PipX and PipY may interact physically with each other, we used the yeast 2-hybrid system. Because artifacts such as occlusion of interaction surfaces by GAL domains occasionally result in false negatives, we tested up to four different pairwise combinations of PipX and PipY fusion proteins, in order to maximize the possibilities of detecting interaction signals. We constructed PipY fusions to N-terminally located GAL4AD (^AD^PipY) or GAL4BD (^BD^PipY) domains as well as PipY (PipY^AD^ and PipY^BD^) and PipX (PipX^BD^) fusions to C-terminally located domains of GAL4 and performed assays with additional PipX (^AD^PipX and ^BD^PipX) and PII (^AD^PII and ^BD^PII) fusions, the later ones to provide positive controls. Interaction signals were detected by the control pairs PII-PII and PII-PipX, but not for PipX-PipX, PipY-PipY or PipX-PipY (**Figure 4C**). While the lack of self-interaction signals agrees with the monomeric nature of both PipX and PipY, the lack of interactions signals from the four PipX-PipY combinations tested indicate that these proteins do not interact in the yeast system.

### Transcript Analysis Reveals Functional Connections between PipY and the Co-regulator PipX

Next, we explored interactions between PipY and PipX in the context of transcriptional regulation. PipX, known as the co-activator of NtcA, is involved in a wider regulatory network, affecting multiple functions in *S. elongatus* (Espinosa et al., 2014) and we wondered whether PipY may play a role in connection with the transcriptional functions of PipX as a global regulator of gene expression. To explore this idea, we compared transcript profiles of WT, *pipX*, *pipY* and *pipXpipY* strains. Because of the polar effects observed at the *pipXY* operon (**Figure 2** and Supplementary Figure S1D), interpretation of orthodox mutant/WT comparisons appears to be straightforward for the double *pipXpipY* mutant but not for the single ones, since the *pipX* null strain accumulates very little PipY protein. However, by performing *pipY/pipXpipY* or *pipX/pipXpipY* comparisons we can focus on the effects provided by the presence of PipX or PipY proteins, even if at a low level, in the absence of the other.

**FIGURE 5:**
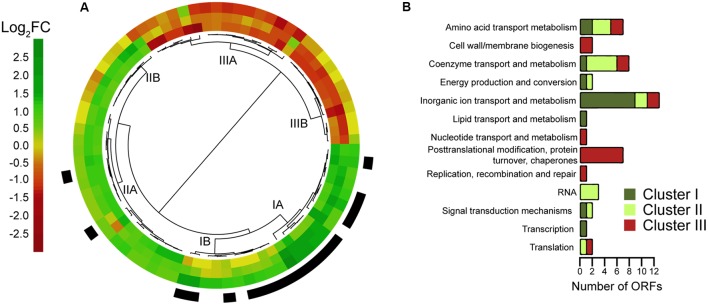
Clustering and functional categories of genes differentially expressed in *pipXY* operon mutants. **(A)** Dendrogram illustrating the classification of 78 differentially expressed genes scoring above the cutoff for a log_2_ fold change of 1 (absolute values) in at least one of the following comparisons: *pipX*/*pipXpipY* (inner ring), *pipY*/*pipXpipY* (middle ring); and WT/*pipXpipY* (outer ring). Expression values are represented using a color code (green: activation; red: repression). NtcA genes [according to (19)] are highlighted in black. **(B)** Distribution of the genes from **(A)** according to their COG (Cluster of Orthologous Groups) classification and manually corrected (see details in Supplementary Table S2). Some genes in Clusters I (7), II (12), and III (9) could not be classified into any COG.

Differentially expressed genes scoring above the cutoff for a log2 fold change in any of the *pipY/pipXpipY*, *pipX/pipXpipY* or WT/*pipXpipY* comparisons were selected. This set of 78 genes was hierarchically clustered according to their expression patterns (**Figure 5A** and Supplementary Table S2). Transcripts up-regulated in any of the three comparisons, for which positive regulation (by PipX and/or PipY) could be inferred, doubled in number to those downregulated. One third (17 out of 51) of the transcripts activated by PipX and/or PipY were NtcA targets, of which 15 clustered together in a group defined by upregulation in the WT/*pipXpipY* comparison [Cluster I, containing only canonical NtcA activated genes (Espinosa et al., 2014)]. Cluster I contains two sub-clusters with expression patterns consistent with PipX and PipY having independent (IA) or additive (IB) effects, respectively. In contrast, Cluster II contained only two NtcA targets, one of which was *gifA*, a gene repressed by NtcA and subjected to complex regulation (Garcia-Dominguez et al., 2000; Galmozzi et al., 2007; Espinosa et al., 2014; Klahn et al., 2015). Cluster II expression patterns are characterized by upregulation in the *pipX/pipXpipY* comparison (Cluster IIA) or in both *pipX/pipXpipY* and *pipY/pipXpipY* comparisons (Group IIB). While the larger Cluster IIA is relatively heterogeneous, Cluster IIB shows a rather uniform pattern consistent with independent activation by PipX and PipY and no activation when both proteins are co-expressed, suggesting that PipX and PipY can interfere with each other functions. Interestingly, the expression patterns of Clusters II and III (were no NtcA targets were found) appear inverted, in particular their more homogeneous subgroups B, with Cluster IIIB characterized by down regulation in both *pipX/pipXpipY* and *pipY/pipXpipY* comparisons.

To further investigate the internal coherence of the groups of genes obtained by hierarchical clustering we followed the COG (cluster of orthologous genes) classification system to assign functions and compare the distribution of COG categories and of genes of unknown function amongst the 3 clusters (**Figure 5B**). Genes of unknown function made similar contributions to the complete *S. elongatus* genome (ca. 37%) and to the 78 genes included in the 3 clusters analyzed here (ca. 36%), but were slightly overrepresented in Cluster II (12 out of 28; ca 43%) and underrepresented in Clusters I (30%) and III (33%). Inorganic ion transport and metabolism genes were overrepresented (16% versus 4% in the complete *S. elongatus* genome), being most abundant in Cluster I (14 out of 23; 39%). The category post-translational modification, protein turnover, chaperones was found exclusively in Cluster III (7 out of 27; 25%), while coenzyme transport and metabolism genes were more represented in Cluster II (5 out of 28; 17%). Amino acid transport and metabolism was the only category appearing in all three clusters with a similar abundance (2 out of 23, 3 out of 28 and 2 out of 27 for Clusters I, II and III, respectively). The remaining categories were represented in just one of the clusters and contained a maximum of 2 genes.

### PipY Overexpression Increases *S. elongatus* Cell Length

**FIGURE 6:**
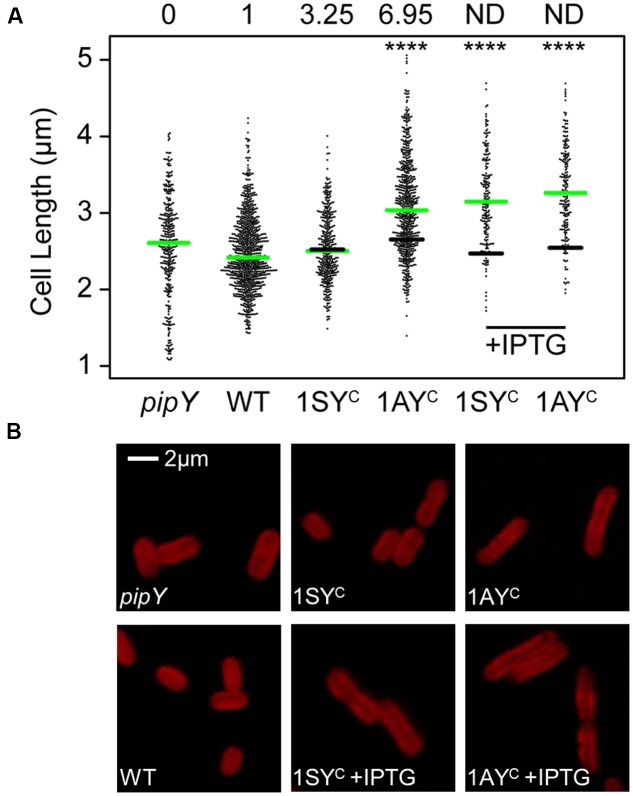
Effects of PipY protein levels on *S. elongatus* cell length. **(A)** Scatterplot of cell lengths for the indicated strains. The median for each strain is indicated with a green line. For 1AY^C^ and 1SY^C^ strains (1^A^Ptrc-PipY and 1^S^Ptrc-PipY, respectively) the black line indicates the median of their respective control strain (1^A^Ptrc and 1^S^Ptrc, respectively). The minimum number of cells measured was *n* = 186. Wilcoxon rank sum test analysis of cell length data produced *p*-values < 0.0001 (^∗∗∗∗^). PipY protein levels relative to that in their control are indicated for each strain on the top. **(B)** Representative cell micrographs from strains in **(A)**. Pictures were taken after 24 h of incubation. Where indicated, IPTG was used at 1mM, final concentration.

The strong *pipY-sepF* synteny in cyanobacteria made us wonder whether *S. elongatus* PipY has a role on the regulation of cell division or cell size. Since *pipY* cells did not show the filamentous phenotypes obtained by depletion of proteins with structural roles in cell division (**Figure 6**), we investigated whether higher levels of PipY would result in a cell division related phenotype. To this end, we used *S. elongatus* strains (Supplementary Figure S3A) expressing *pipY* from a Ptrc promoter in an ectopic location (NSI). This allowed us to increase the levels of PipY either constitutively or to much higher levels after IPTG induction. Strains 1^S^Ptrc-PipY and 1^A^Ptrc-PipY (in reference to selection markers Sm^R^ or Apr^R^, respectively) were tested alongside control strains 1^S^Ptrc and 1^A^Ptrc. As shown in **Figure 2**, strains 1^S^Ptrc-PipY and 1^A^Ptrc-PipY allowed moderate increases of PipY levels (∼4-fold and ∼7-fold over the WT levels, respectively) in the absence of IPTG.

Cells from exponentially growing cultures were observed by Laser Scanner Confocal Microscopy and their length measured. As shown in **Figure 6**, elimination of PipY or a ∼4-fold increase over the WT levels did not alter cell dimensions in a significant way, while larger increases in the PipY levels correlated with larger cells. A ∼7-fold increase in PipY resulted in a 14.4% of increase in the average cell length of *S. elongatus*. Further overexpression in the presence of IPTG resulted in a 28% increase in the average cell length.

## Discussion

Our genetic analyses in *S. elongatus* show that *pipY* is a typical member of the COG0325 family of PLP-binding proteins recruited to the cyanobacterial nitrogen regulatory network. Phenotypic analyses with the *pipY* mutant demonstrated the involvement of PipY in amino/keto acid and PLP homeostasis. In addition, work with *pipX* and *pipY* mutant and derivative strains revealed gene interactions in the contexts of survival to PLP targeting antibiotics and of transcriptional regulation, placing PipY in the same genetic pathway as PipX, a protein interacting with 2-OG sensors in cyanobacteria.

Several lines of evidence support the universality of COG0325 functions, suggesting a regulatory rather than an enzymatic function for COG0325 proteins. The pyridoxine toxicity phenotype reported for *E. coli yggS* (Prunetti et al., 2016) could be rescued by heterologous expression of PROSC (Darin et al., 2016) and here we show that the *S. elongatus pipY* mutant is also hypersensitive to pyridoxine. Importantly, despite the fact that the Val excretion phenotype reported for the *E. coli* MG1655 *yggS* mutant (Ito et al., 2013) was not confirmed for *E. coli* BW25133 *yggS* (Prunetti et al., 2016) it could nevertheless be rescued by expressing the plant or human (named PROSC) COG0325 proteins (Ito et al., 2013). Thus, distantly related COG0325 proteins can contribute to metabolic homeostasis in different cell types, rescuing species (or even strain) -specific defects. It is worth noting that the lack of *in vitro* evidence of amino acid racemase, decarboxylase, deaminase or transaminase activities from the *E. coli*, *B. subtilis* or human proteins (Ito et al., 2013), and the lack of viability of *alr* mutants in *S. elongatus*, even in the presence of increased levels of PipY, argue against these proteins having enzymatic functions.

The high reactivity of PLP implies that cells must have mechanisms to keep the intracellular level of PLP low while supplying enough PLP for the newly synthesized apo-B6 enzymes to become active. Amongst these mechanisms are the effective feedback inhibition by PLP and its tight binding to the producing enzymes PNP oxidase, PL kinase and PLP synthase [(Ghatge et al., 2012) and references within] and the ability of PLP-dependent enzymes to trigger product release from the PLP synthase (Moccand et al., 2011). To cope with the PLP derived toxicity and optimize the delivery of the cofactors to PLP-apoenzymes, some PLP-binding proteins would have evolved to act as PLP reservoirs and PLP delivering modules for the essential apo-enzymes. It is worth noting that, in contrast to the situation in type III enzymes, the PLP cofactor of COG0325 proteins is solvent exposed [discussed in Ito et al. (2013)], and thus appears to be appropriately placed for the proposed roles on PLP homeostasis.

The synthetic lethality observed here between *S. elongatus pipY* and *cysK* has a precedent in the conditional synthetic lethality reported for *E. coli yggS* and *glyA* (Nichols et al., 2011; Prunetti et al., 2016). The inferred functional redundancy between COG0325 proteins and two different PLP-holoenzymes strongly suggests that CysK and GlyA also contribute to PLP homeostasis in *S. elongatus* and *E. coli*, respectively. Functional redundancy amongst PLP-holoenzymes appears to occur even in organisms with relatively small genomes, as *S. elongatus*, where out of the 41 PLP-binding protein sequences found (Percudani and Peracchi, 2009), 11 corresponded to non-essential and 6 to beneficial genes (their inactivation slows growth of cultures) under standard photoautotrophic conditions (Guerreiro et al., 2014; Rubin et al., 2015). Functional redundancy between the regulatory COG0325 proteins and PLP-holoenzymes, revealed by synthetic lethality in two distantly related bacteria, suggest that at least some of the multiple PLP-containing proteins expressed in leaving cells participate in PLP homeostasis. It also explains that, despite the universality of the PLP-derived challenges in all types of cells, COG0325 proteins are neither essential (in the so far characterized systems), nor ubiquitous (Prunetti et al., 2016).

The main mechanism of resistance to DCS in bacteria is overexpression of the essential protein alanine racemase (Caceres et al., 1997). In contrast, PipY overexpression did not increase resistance to DCS or BCDA in *S. elongatus*. This finding, and the implication of PipX in survival to DCS indicates a very different basis for the protective role of PipY against DCS, supporting the idea that PipY protects the essential and high affinity targets against DCS indirectly, by affecting the activity of PLP holo-enzymes. In this context, there are precedents for regulatory genes involved in basic metabolic processes whose inactivation alters bacterial susceptibility to antibiotics (Martinez and Rojo, 2011). In *S. elongatus* PipX and/or PipY would contribute to increasing the intracellular levels of DCS antagonists, that is, alanine or related amino acids. It is worth noting that inactivation of L-alanine dehydrogenase is also a mechanism for DCS resistance (Desjardins et al., 2016) and that nitrogen specific responses have been implicated in the control of alanine metabolism (Lahmi et al., 2006).

Given the regulatory complexity involving PLP-holoenzymes and central metabolism, it now seems naive to expect that COG0325 deficiency caused accumulation or depletion of the same metabolite(s) in different experimental conditions or cell types. However, the prediction is that COG0325 defective mutants would be impaired in the regulation of biological pathways or processes responsive to amino/keto acids. One of such processes is the nitrogen regulatory network of cyanobacteria, which is integrating and transmitting signals of the 2-OG levels, the main indicator of the nitrogen/carbon balance in these organisms.

Two lines of results place PipX and PipY within the same genetic pathway. In particular, both proteins make contributions to resistance to PLP-targeting antibiotics DCS or BCDA and to expression of a common set of transcripts. The observed genetic interactions between PipY and PipX are best explained by PipY affecting the levels of the amino/keto acids relevant in those biological pathways and, although physical interactions between PipX and PipY proteins in *S. elongatus* can not be ruled out at this stage, exhaustive yeast two-hybrid interaction assays did not support it.

**FIGURE 7:**
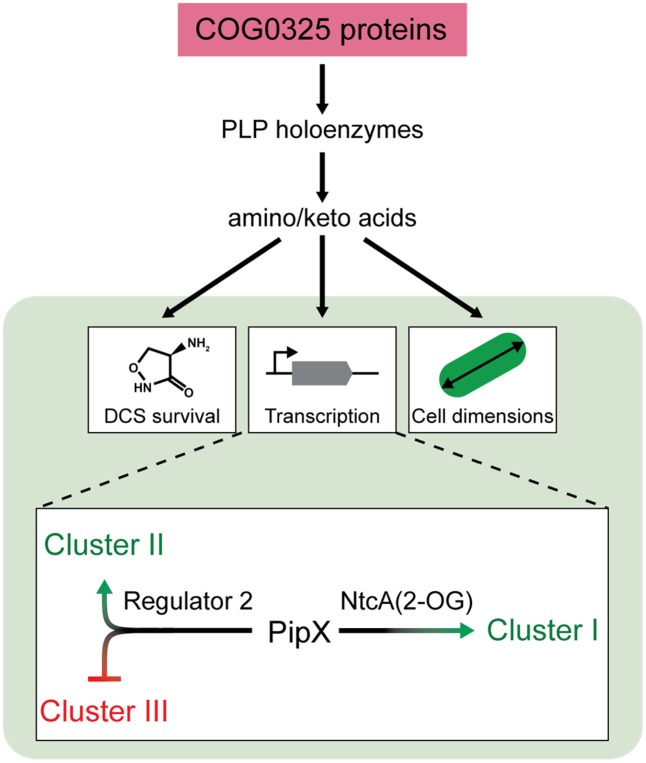
Model for universal COG0325 functions and processes regulated by PipY in cyanobacteria. COG0325 proteins, functioning as a PLP storage and delivery module, influence the activity of PLP-holoenzymes, which in turns affect the amino/keto acid pool and the processes responsive to (some of) these metabolites. In cyanobacteria (lower part of the figure, shadowed green), PipY would contribute to survival in the presence of DCS or other antibiotics targeting PLP-holoenzymes, affect expression of groups of genes, and alter cell dimensions under particular nutrient conditions. Gene targets would be modulated by PipY and PipX, either via NtcA (Cluster I), whose activity is known to be stimulated by PipX in the presence of 2-OG, or by Regulator 2 (Clusters II and III). See text for additional details.

The similar effects of PipX and PipY on NtcA targets (Cluster I in **Figure 5**) suggest that PipY may have a positive effect on the intracellular levels of 2-OG under the experimental conditions used here. The subdivision of Cluster I transcripts into two sub-clusters with expression patterns suggestive of PipX and PipY having independent (IA) or additive (IB) effects, respectively, can be rationalized on the bases of the affinity of NtcA for its different binding sites (Espinosa et al., 2007; Forcada-Nadal et al., 2014). NtcA targets from Cluster IB would provide weaker NtcA binding and thus greater dependency on both PipX and PipY. The transcriptome-based genetic analysis performed here supports previous work indicating that PipX modulates a large set of genes that also include NtcA-independent target genes (Espinosa et al., 2014; Labella et al., 2016) and further suggest that at least two types of transcriptional complexes are co-regulated by PipY and PipX (**Figure 7**). One would correspond to the already characterized NtcA(2-OG)-PipX complexes and the other one, defined by Clusters II and III, would require the implication of a second transcriptional regulator exerting opposite effects in Clusters II and III (“Regulator 2” in **Figure 7**). PlmA, the only other transcriptional regulator known to interact with PipX (Labella et al., 2016), is so far the best candidate for Regulator 2. It follows that the expression of Clusters II and III could also be affected by 2-OG or related metabolite(s) whose levels would be influenced by PipY.

Synteny between *ylmE*, the gram-positive ortholog of *pipY*, and cell division genes or cell division clusters has been the subject of discussions (Gola et al., 2015; Prunetti et al., 2016), but there are only two reports assigning cell size or cell wall related phenotypes to *ylmE* null or overexpressing strains. *Streptococcus pneumoniae ylmE* cells were slightly larger (Fadda et al., 2003), while a *B. subtilis* strain overexpressing YlmE was blocked in biofilm formation (Kolodkin-Gal et al., 2010). In addition, no phenotype, that is, normal cell dimensions under standard culture conditions have been reported for *E. coli yggS* (Prunetti et al., 2016) and found here for *S. elongatus pip*Y. However, genetic alterations causing more drastic alterations of amino/keto acid homeostasis, such as presumably double mutations (*yggS glyA*) in *E. coli* (Prunetti et al., 2016) and overexpression of PipY increased cell length up to 23% or 28%, respectively. These findings support the notion that perturbations of the amino/keto acid pool may result in the accumulation of metabolic signals controlling processes related to cell wall metabolism or cell size. In this context, the best characterized example of a nutrient-dependent pathway coordinating cell division and cell size with growth rate (using UDP-glucose as a molecular proxy) is that of the Gram-positive model organism *B. subtilis* (Chien et al., 2012). In the case of *S. elongatus*, a link between particular nutrient conditions (combined phosphorus limitation and abundancy of reduced nitrogen) and morphological flexibility has been established (Goclaw-Binder et al., 2012), raising questions on whether this phenomenon relays on amino/keto acids signals.

In summary, the results presented here provide important insights into the COG0325 family of proteins and the universality of their functions, clearly interconnected with the activity of PLP-holoenzymes. Work with PipY, the cyanobacterial member of this widespread family, confirms the conserved roles in vitamin B6 and amino/keto acid homeostasis and extends our functional understanding of COG0325 proteins. We also provide genetic evidence for the recruitment of PipY into the 2-OG-dependent nitrogen interaction network. This work emphasizes the regulatory importance of COG0325 proteins in central metabolism and suggest their implication in the metabolic-dependent coordination of cell size.

## Author Contributions

Designed research: AC, JL, RC, JE, and VR. Performed research: JL, RC, JE, VR, and AF-N. Analyzed data: AC, JL, RC, JE, AF-N, and VR. Wrote the paper: AC. Contributed new reagents or analytic tools: JL.

## Conflict of Interest Statement

The authors declare that the research was conducted in the absence of any commercial or financial relationships that could be construed as a potential conflict of interest.

## Footnotes

1 http://eggnogdb.embl.de

2 https://www.ebi.ac.uk/Tools/msa/clustalo/

3 https://dfgm.ua.es/genetica/investigacion/cyanobacterial_genetics/Resources.html

4 http://www.genome.jp/kegg/
