# Supplementary material for: PipY, a Member of the Conserved COG0325 Family of PLP-Binding Proteins, Expands the Cyanobacterial Nitrogen Regulatory Network
Source: Front Microbiol. 2017 Jul 11;8:1244. doi: 10.3389/fmicb.2017.01244 (PMC5504682; doi:10.3389/fmicb.2017.01244)
Supplement: Supplementary file 1 [file Table_1.DOCX]

**Table S1**. **Oligonucleotides**

| **Oligonucleotide name** | **Oligonucleotide sequence (5’-3’)** |
| --- | --- |
| PipX-Cm-1F | TGCACTTGTTACAGAACCCTAGCGAGCACTTGTCCTGGTTCCACTTGAGGATCGATTCCCCCGAGTAATGGAGAAAAAAATCACTGGATA |
| PipX-Cm-1R | GGCAGTTGCGATCGGAGACTGGCCAACCGCTCGGCAATTTGGGCCATGCCTGCCTCTGACTCCCTAGCTGGCTACGCCCCGCCCTGCCAC |
| 2060-Cm-1F | GGAATACAACCAGCTGCAGCAAGTCTTCAAACAAACCTTTCTGTAGCCAGCTAGGGAGTCAGAGGCAGGCATGGAGAAAAAAATCACTGGATA |
| 2060-Cm-1R | GCACCTGAAATGGCAATTGCTAGGCCAGTTCTAAGACGTAAAGGTTTGTTAACTATGGCCAAGACTAGCTGGCTACGCCCCGCCCTGCCAC |
| ADCseq-F | GTCATTGTTCTCGTTCCCTTT |
| GBT-2R | CCTACAGGAAAGAGTTACTC |
| PipXQ34A-F | CGCCACTCTTTATGCTGCGCGCCTCTTTTTTCTCG |
| PipXQ82A-R | CAGAAAGGTTTGTTTGAAGACCGCCTGCAGCTGGTTGTATTCCTG |
| PipXF38A-F | GCTCAGCGCCTCTTTGCTCTCGTAGCCTTTGATGC |
| PipX-4F | CAAATTGCCGAGCGGTTGGC |
| 2059-R | GGGGTCGGAGTCGCAGTCCGGCC |
| 2059-F | GCCCCAGGCCATTCAGGCTTTGCGCG |
| PipX-5R-X | CAGCCCGCAAATCAGCAG |
| inter2060-1F | GATCGGAATTCCCAGCTAGGGAGTCAGAGG |
| PipXXinact-F | CAAGCGAATTCGGCCCGCTAGCTGC |
| PipXXinact-R | GCTGTCTCGAGCGGGAACTGGTTTCG |
| rnpB-R | TAAGCCGGGTTCTGTTCTCT |
| rnpB-F | GTGAGGAGAGTGCCACAGAA |
| PTRC99Aseq | GCCGACATCATAACGG |
| NSI-1F | CGACATCTTCCTGCTCCAG |
| 7942NSIA-F | GACGGGTACCTCTGCTGG |
| NSI-1R | TGCCTGAAAGCGTGACGAGC |
| ALREcoRI-F | TACATGAATTCCGAATCACCGTCGGA |
| ALRBamHI-R | CTAAGGGATCCGGGAGAGACTGTCCCA |
| nat1ALR-F | TGAAACCGATTGATCCCCAACCAACTCCGCGAGCAAACAAGCAAAGGAA |
| nat1ALR-R | TGACGACTGCCATCAATTCACAACTGGGTGCGGCCTTAGGGGCAG |
| Apramicyn-1F | GAAAAGCTTCACGAACCCTTTGGC |
| Apramicyn-1R | ATGCATGCAGGCTGGAGCTGCTTCGAAG |
| PipXOV2F | GAGAATTCGCTTCCGAGAACTACC |
| PipX3R | TCGATCGGATCCTGGCCAACC |
| ORF2060-4F | GATCGGAATTCATGGCCCAAATTGC |
| 2060-3R | GATCGGTCGACTTACAGCGATCGCGGCCCA |
| cysK-1F | CCTACAGCTAGGGTTG |
| cysK-1R | CTGATAGTGATCCGCC |
| PipX-ADC-F | AGCATACAATCCAAGATGGCTTCCGAGAACTACCTCAACCATCCC |
| PipX-ADC-R | CGCTTTATCCATCTTTGCAAAGGCCAGAAAGGTTTGTTTGAAGACTTGCTGCAGC |
| PipX-BDC-F | GCTTGAAGCAAGCCTCGATGGCTTCCGAGAACTACCTCAACCATCCC |
| PipX-BDC-R | CAGTAGCTTCATCTTTCGCAGAAAGGTTTGTTTGAAGACTTGCTGCAGC |
| PipY-ADC-F | AGCATACAATCCAAGATGGCCCAAATTGCCGAGCGGTTGG |
| PipY-ADC-R | CGCTTTATCCATCTTTGCAAAGGCCAGCGATCGCGGCCCAAATAACTGGGT |
| PipY-BDC-F | GCTTGAAGCAAGCCTCGATGGCCCAAATTGCCGAGCGGTTG |
| PipY-BDC-R | CAGTAGCTTCATCTTTCGCAGCGATCGCGGCCCAAATAACTGGGT |
| ADC-F | CAAGCTATACCAAGCATACAATCCAAGATG |
| ADC-R | GGAATTAATTCCGCTTTATCCATCTTTGCAAAGGC |
| BDC-F | CAACTCCAAGCTTGAAGCAAGCCTCGATG |
| BDC-R | CGATAGAAGACAGTAGCTTCATCTTTCG |
| PIXX-F | CAGAGGAATTCATGGCCCAAATTGCCG |
| PIXX-R | TATGGGTCGACTTACAGCGATCGCGGCCC |
| PipX126-F | TAAAAACTAGCCGCCCTTGC |
| qPCR2060-1R | GAATGAATCCAGTCGAAATGCT |
| Syn2062-1F | GGCCTGCCGAAGGAGCAGTGGTACG |
