# Supplementary material for: PipY, a Member of the Conserved COG0325 Family of PLP-Binding Proteins, Expands the Cyanobacterial Nitrogen Regulatory Network
Source: Front Microbiol. 2017 Jul 11;8:1244. doi: 10.3389/fmicb.2017.01244 (PMC5504682; doi:10.3389/fmicb.2017.01244)
Supplement: Supplementary file 2 [file Table_2.DOCX]

**Table S2*.* List of differentially expressed genes.** Genes (log_2_FC above 1 in at least one of the comparisons) with their COG assignment and named with their id. Strain code: *pipX* (X), *pipY* (Y) and *pipXpipY* (XY).

| Cluster | Gene id | Log_2_FC | | | COG |
| --- | --- | --- | --- | --- | --- |
|  |  | X/XY | Y/XY | WT/XY |  |
| IA | Synpcc7942_0841 | 1,41 | 1,22 | 1,2 | Inorganic ion transport and metabolism |
|  | Synpcc7942_0206 | 1,32 | 1,05 | 1,13 | Coenzyme transport and metabolism |
|  | Synpcc7942_1673 | 1,27 | 1,46 | 0,93 | Not in COGs |
|  | Synpcc7942_0763 | 1,26 | 1,36 | 0,81 | Transcription |
|  | Synpcc7942_1032 | 0,9 | 1,53 | 1,32 | Function unknown |
|  | Synpcc7942_1713 | 0,84 | 1,39 | 1,13 | Lipid transport and metabolism |
|  | Synpcc7942_0891 | 0,76 | 1,33 | 0,77 | Not in COGs |
|  | Synpcc7942_B2650 | 1,87 | 1,89 | 0,77 | Not in COGs |
|  | Synpcc7942_0127 | 0,49 | 1,61 | 1,5 | Signal transduction mechanisms |
|  | Synpcc7942_2279 | 0,52 | 1,43 | 1,47 | Inorganic ion transport and metabolism |
|  | Synpcc7942_1240 | 0,49 | 1,54 | 1,75 | Inorganic ion transport and metabolism |
|  | Synpcc7942_1036 | 1,31 | 1,74 | 1,98 | Not in COGs |
|  | Synpcc7942_2107 | 0,71 | 2,23 | 3,6 | Inorganic ion transport and metabolism |
| IB | Synpcc7942_2173 | 0,39 | 0,42 | 1,02 | Energy production and conversion |
|  | Synpcc7942_1033 | 0,13 | 0,74 | 1,1 | Amino acid transport and metabolism |
|  | Synpcc7942_2156 | -0,02 | 0,42 | 1,21 | Amino acid transport and metabolism |
|  | Synpcc7942_1239 | -0,02 | 0,35 | 1,13 | Inorganic ion transport and metabolism |
|  | Synpcc7942_2157 | 0,02 | 0,51 | 1,01 | Not in COGs |
|  | Synpcc7942_B2662 | 0,13 | 0,13 | 1,24 | Not in COGs |
|  | Synpcc7942_1238 | 0,29 | 0,22 | 1,14 | Inorganic ion transport and metabolism |
|  | Synpcc7942_1689 | 0,01 | -0,08 | 1,28 | Inorganic ion transport and metabolism |
|  | Synpcc7942_0442 | -0,2 | 0,7 | 1,96 | Inorganic ion transport and metabolism |
|  | Synpcc7942_2105 | 0,04 | 0,44 | 1,73 | Inorganic ion transport and metabolism |
| IIA | Synpcc7942_0497 | 1,31 | 0,75 | 0,66 | Not in COGs |
|  | Synpcc7942_2540 | 1,31 | 0,84 | 0,65 | Inorganic ion transport and metabolism |
|  | Synpcc7942_1070 | 1,38 | 0,96 | 0,83 | General function prediction only |
|  | Synpcc7942_2172 | 1,09 | 0,82 | 0,63 | Not in COGs |
|  | Synpcc7942_0292 | 1,11 | 0,73 | 0,69 | Coenzyme transport and metabolism |
|  | Synpcc7942_0103 | 0,88 | 0,52 | 1,01 | General function prediction only |
|  | Synpcc7942_2527 | 0,79 | 0,71 | 1,19 | Amino acid transport and metabolism |
|  | Synpcc7942_2466 | 1,13 | 0,2 | 0,55 | Signal transduction mechanisms |
|  | Synpcc7942_2175 | 1,32 | -0,48 | 0,55 | Inorganic ion transport and metabolism |
|  | Synpcc7942_0768 | 1,17 | 0,87 | 0,35 | Coenzyme transport and metabolism |
|  | Synpcc7942_1290 | 1,17 | 0,88 | 0,4 | Not in COGs |
|  | Synpcc7942_0769 | 1,02 | 0,78 | 0,39 | Not in COGs |
|  | Synpcc7942_0900 | 1 | 1,03 | 0,44 | Amino acid transport and metabolism |
|  | Synpcc7942_1072 | 1,1 | 1,05 | 0,48 | Coenzyme transport and metabolism |
|  | Synpcc7942_R0001 | 1,14 | 1,2 | 0,41 | RNA |
|  | Synpcc7942_R0047 | 0,82 | 1,03 | 0,42 | RNA |
|  | Synpcc7942_R0023 | 0,72 | 1,01 | 0,49 | RNA |
|  | Synpcc7942_1422 | 0,8 | 1,05 | 0,23 | Energy production and conversion |
|  | Synpcc7942_2042 | 1,05 | 0,78 | -0,02 | Not in COGs |
|  | Synpcc7942_2082 | 1,05 | 0,79 | 0,04 | Translation |
| IIB | Synpcc7942_2241 | 0,77 | 1,07 | -0,28 | Not in COGs |
|  | Synpcc7942_2514 | 0,8 | 1,16 | -0,16 | Amino acid transport and metabolism |
|  | Synpcc7942_0162 | 0,67 | 1,27 | -0,41 | Not in COGs |
|  | Synpcc7942_0259 | 0,77 | 1,05 | -0,06 | Not in COGs |
|  | Synpcc7942_0491 | 0,82 | 1,04 | 0,02 | Not in COGs |
|  | Synpcc7942_0163 | 0,95 | 1,43 | -0,03 | Coenzyme transport and metabolism |
|  | Synpcc7942_1833 | 0,89 | 1,33 | -0,13 | Coenzyme transport and metabolism |
|  | Synpcc7942_2126 | 0,67 | 1,02 | -0,8 | Not in COGs |
| IIIA | Synpcc7942_2480 | -1,85 | -0,06 | -0,52 | Posttranslational modification, protein turnover, chaperones |
|  | Synpcc7942_2482 | -1,43 | -0,24 | -0,62 | Not in COGs |
|  | Synpcc7942_B2649 | -1,19 | -0,64 | -0,51 | Not in COGs |
|  | Synpcc7942_1682 | -1,19 | -0,39 | -0,34 | Posttranslational modification, protein turnover, chaperones |
|  | Synpcc7942_1681 | -1,66 | -0,14 | 0,5 | Inorganic ion transport and metabolism |
|  | Synpcc7942_2481 | -2,64 | -0,51 | -1,14 | Not in COGs |
|  | Synpcc7942_1663 | -0,5 | -0,49 | -1,06 | Not in COGs |
|  | Synpcc7942_1043 | -0,57 | -0,54 | -1,28 | Coenzyme transport and metabolism |
|  | Synpcc7942_1683 | -0,77 | -0,49 | -1,13 | Amino acid transport and metabolism |
|  | Synpcc7942_B2633 | -0,74 | -0,59 | -1,24 | Not in COGs |
|  | Synpcc7942_1997 | -0,63 | -0,25 | -1,11 | Not in COGs |
|  | Synpcc7942_0454 | -0,94 | -0,32 | -1,03 | Coenzyme transport and metabolism |
|  | Synpcc7942_0043 | -1,05 | -0,43 | -0,82 | Not in COGs |
|  | Synpcc7942_1120 | -1,35 | -1 | -1,09 | Not in COGs |
|  | Synpcc7942_0894 | -0,97 | -0,97 | -1,44 | Amino acid transport and metabolism |
|  | Synpcc7942_2026 | -0,21 | 0,47 | -1,39 | Cell wall/membrane biogenesis |
| IIIB | Synpcc7942_2468 | -1,15 | -1,04 | -0,29 | Posttranslational modification, protein turnover, chaperones |
|  | Synpcc7942_0630 | -1,1 | -0,94 | -0,25 | Cell wall/membrane biogenesis |
|  | Synpcc7942_1661 | -1,15 | -0,85 | 0,03 | Not in COGs |
|  | Synpcc7942_2314 | -0,96 | -1,1 | 0,02 | Posttranslational modification, protein turnover, chaperones |
|  | Synpcc7942_0685 | -1,1 | -1,23 | -0,08 | Posttranslational modification, protein turnover, chaperones |
|  | Synpcc7942_0486 | -0,78 | -1,02 | -0,24 | Nucleotide transport and metabolism |
|  | Synpcc7942_1813 | -0,93 | -1,1 | -0,18 | Posttranslational modification, protein turnover, chaperones |
|  | Synpcc7942_2313 | -1,31 | -1,43 | 0,09 | Posttranslational modification, protein turnover, chaperones |
|  | Synpcc7942_0112 | -0,48 | -1,29 | 0,02 | Replication, recombination and repair |
|  | Synpcc7942_2085 | -0,43 | -1,24 | -0,01 | Inorganic ion transport and metabolism |
|  | Synpcc7942_2235 | -0,57 | -1,07 | 0,33 | Translation |
