# Supplementary material for: PipY, a Member of the Conserved COG0325 Family of PLP-Binding Proteins, Expands the Cyanobacterial Nitrogen Regulatory Network
Source: Front Microbiol. 2017 Jul 11;8:1244. doi: 10.3389/fmicb.2017.01244 (PMC5504682; doi:10.3389/fmicb.2017.01244)
Supplement: Supplementary file 3 [file Image_1.pdf]

Fig. S1

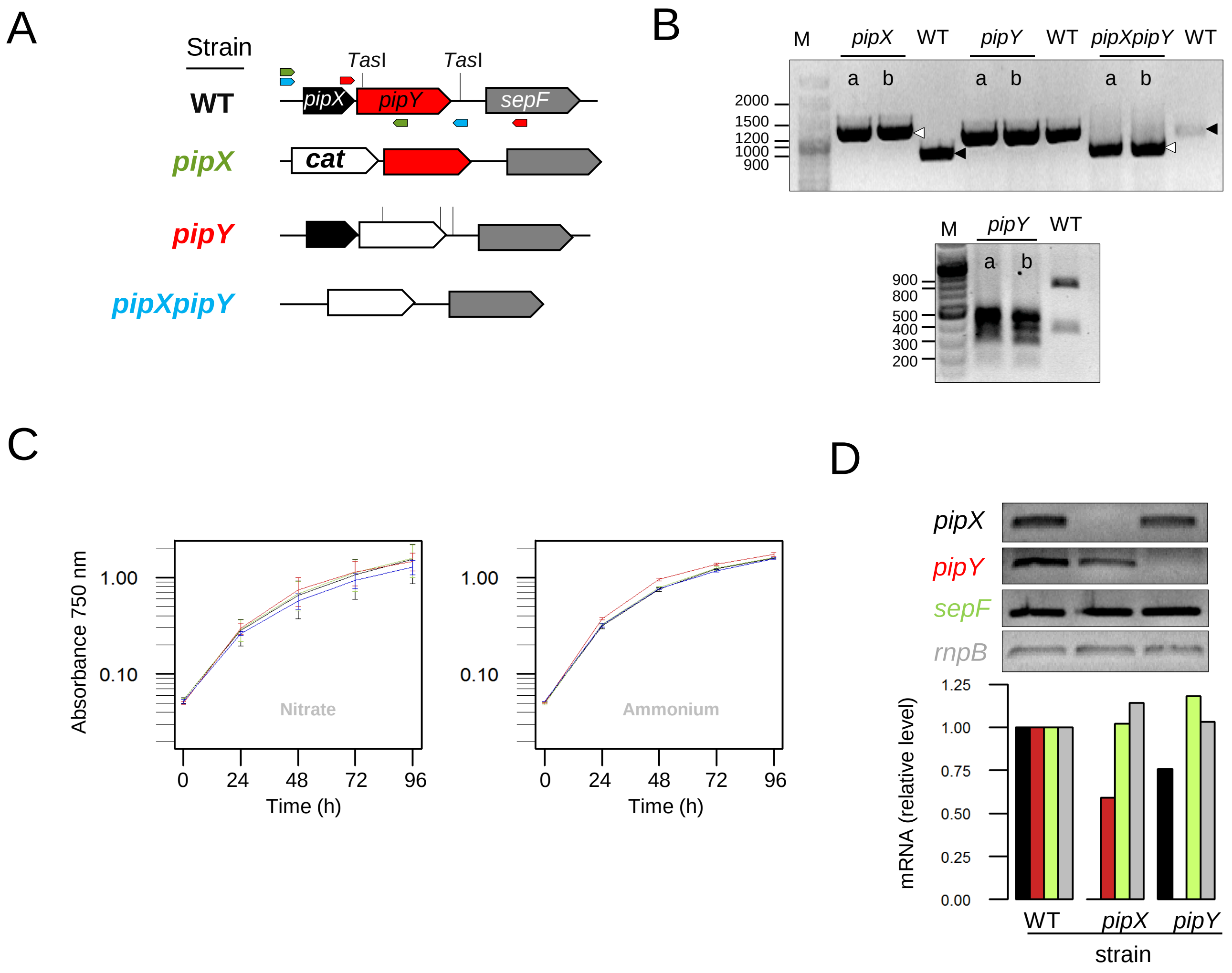

Figure S1. Construction of *S. elongatus pipX, pipY* and *pipXpipY* null mutants, effects of mutations in growth rate and transcript levels. (A) Schematic representation of the relevant *S. elongatus* region and strategy to precisely replace *pipX* and/or *pipY* ORFs by the *cat* gene. The same color code is used for each gene(s) inactivated and the pair of primers (arrows) used to analyze the segregation of the null alleles. Relevant *Tas*I sites are indicated with vertical lines. **(B)** Analysis of two independent mutant clones (a and b) for each strain. Upper panel, amplified wild-type (closed arrowhead) or null (open arrowhead) alleles are indicated. Bottom panel, *Tas*I-RFLP analysis of amplified wild type *pipY* and null alleles (upper panel) present in the WT and *pipY* mutant, respectively. Reference size bands are indicated on the left (lane M). **(C)** Growth curves of WT and mutant derivatives in medium with either nitrate or ammonium. Mean values with standard deviation of two independent experiments in each condition are plotted**. (D)** Quantification of the band intensity of transcripts in the RT-PCR by ImageJ software. Bar values are presented as the ratio of the intensity of the corresponding transcript in the indicated strain relative to the intensity found in the WT. Intensity of quantified bands in the WT were set arbitrarily to 1. The same color code is used for each transcript in the bar histogram and the RT-PCR panel. Data correspond to one RNA extraction and *rnpB* is used as a loading control.

Fig. S2

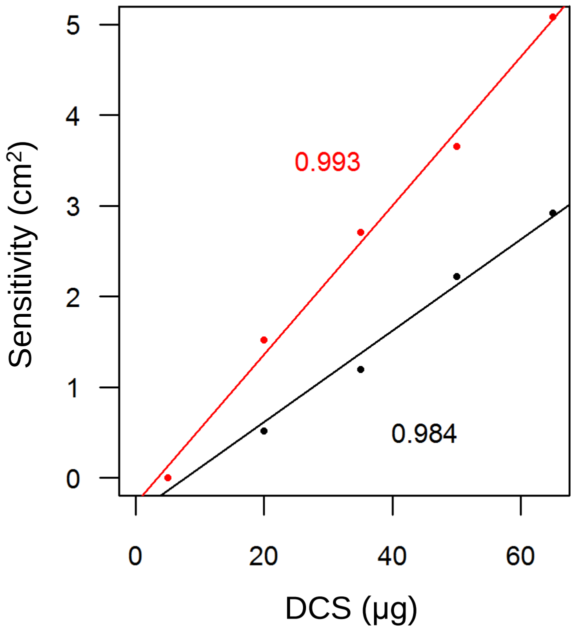

Figure S2. Correlation between sensitivity and the amount of DCS added in sensitivity assays. *S. elongatus* disc diffusion assays showing correlation between sensitivity (as squared radius of the growth inhibition halo) and the amount of DCS spotted on the disc for wild type (black) and *pipY* mutant (red). Adjusted r-squared of the fitted lines is shown with the same color code. Mean of two replicates is plotted.

Fig. S3

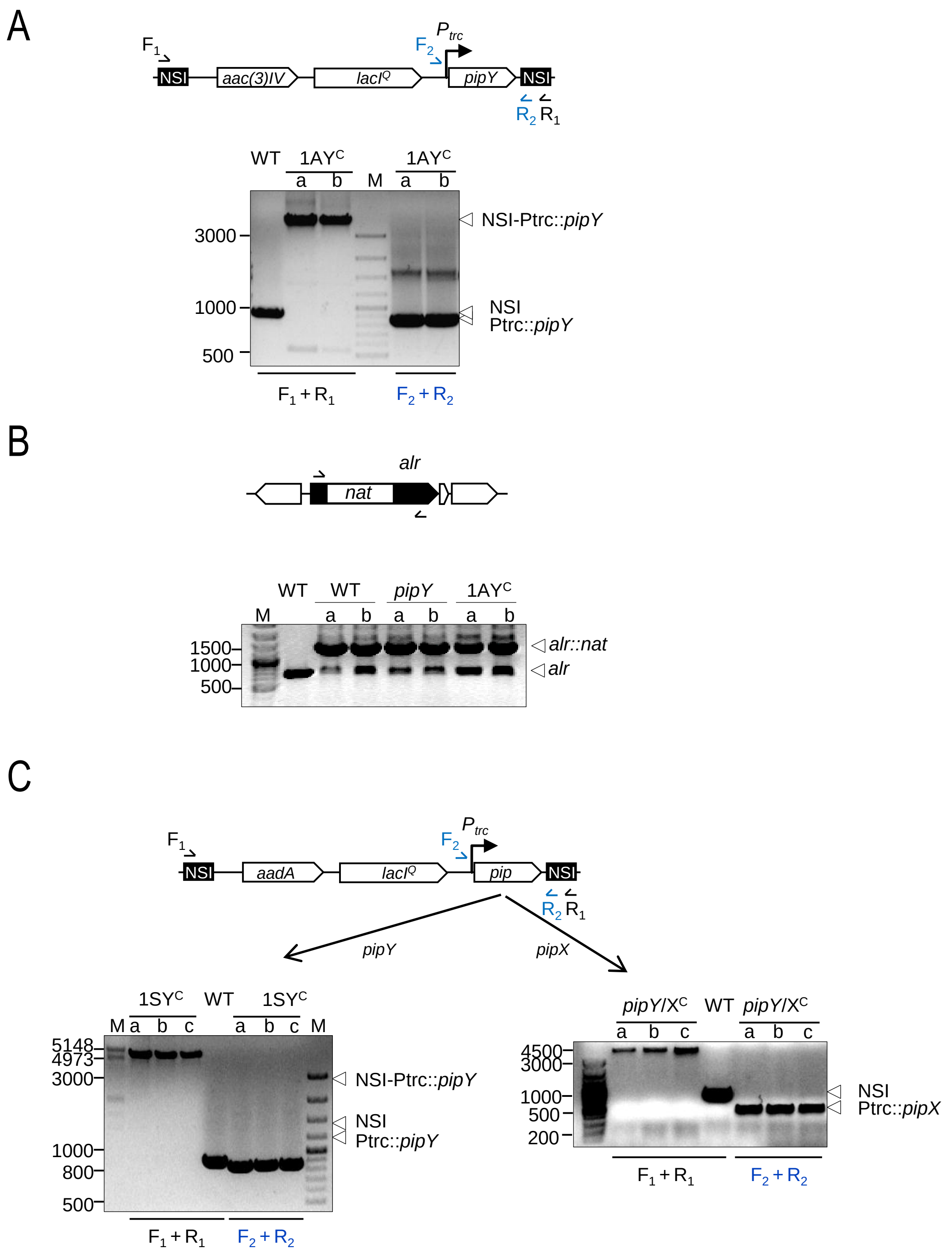

Figure S3. Construction and analysis of *S. elongatus* strains derivatives. Schematic representation of the relevant *S. elongatus* region is always above the gels from PCR analysis of two or three independently transformed clones (a, b, c) to verify constructions. **(A, C)** Strategies to construct P*trc* derivates (*pip* refers to *pipX* or *pipY* as indicated), with either *aac(3)IV* ($1AY^{C}$) or *aadA* ($1SY^{C}$) gene into neutral site I (NSI). **(B)** Strategy to inactivate *alr* in WT, *pipY,* and 1^{A}Ptrc-PipY ($1AY^{C}$) backgrounds. In all cases the positions of relevant primer pairs (arrows) are indicated. Reference size bands and amplified alleles are always shown to the left and right, respectively. M: Size marker lane.

Fig. S4

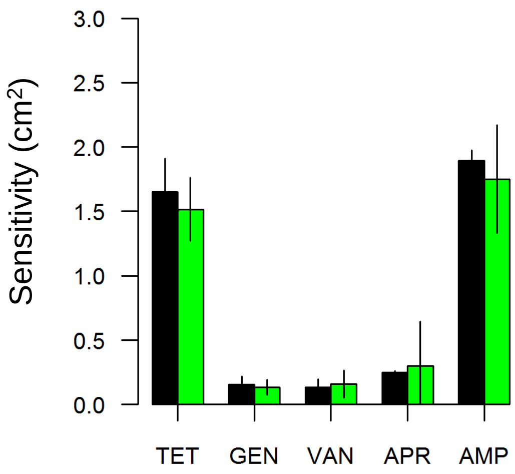

Figure S4. Sentitivity of *pipX* mutant to different antibiotics. Sensitivity levels of WT (black) and *pipX* (green) for the indicated antibiotics shown as bar histogram of means and standard deviations (SD) for at least two independent experiments. Other details as in **Figure 3**.
